# Society Proceedings

**Published:** 1903-05

**Authors:** 


### SOCIETY PROCEEDINGS.

### RHODE ISLAND VETERINARY MEDICAL ASSOCIATION.

A meeting of the veterinary surgeons of Rhode Island was held in the Hotel Dorrance, January 27th, for the purpose of organizing a Rhode Island Veterinary Medical Association, and the promoters of the new organization are said to be planning to secure the enactment of a State law regulating the practice of veterinary medicine and surgery. There are twenty or more regularly graduated veterinarians in the State. It is proposed to eventually secure the passage of a law regulating practice, and the Legislature will be asked to enact a law prohibiting the practice of veterinary medicine and surgery by those not possessed of a diploma as evidence of proper training, unless they have regularly engaged in practice as veterinarians for a period of five years preceding the passage of the proposed act.

The meeting was called to order by Dr. L. T. Dunn. One of the first matters of business was the election of officers, which resulted as follows: President, A. T. Parker; First Vice-President, L. T. Dunn; Second Vice-President, J. S. Pollard; Secretary, T. E. Robinson; Treasurer, J. T. Cunningham; Executive Committee, Drs. Dunn, Fry, Richardson, Bertran, Monahan, McLaughlin, and Tucker.

The members of the Executive Committee will draw up a set of by-laws to be presented at the next meeting of the organization. At present the social features of the Association are said by members to be the principal benefits to be derived. No legislative action was taken at this meeting.

Governor Garvin was, as a practising physician, made an honorary member of the Society.

L. T. DUNN, D. V. S.

### PASSAIC COUNTY VETERINARY MEDICAL ASSOCIATION.

The regular monthly meeting of the Passaic County Veterinary Medical Association was held at 169 Paterson Street, Paterson, N. J., on Tuesday evening, February 3, 1903, with Dr. William Herbert Lowe, President, in the chair.

On roll-call the following members answered to their names: Drs. William J. Reagan, M. A. Pierce, T. J. Cooper, Wm. H. H. Doty, David Machan, John H. Degraw, Wm. Herbert Lowe, of Paterson; William C. Berry, of Haskell, and J. Payne Lowe, of Passaic.

Dr. David Machan was chosen Secretary *pro tem.*

The minutes of the last regular meeting were read and approved.

Dr. Cooper moved that the Association provide for a substitute for the relief of any member when needing the services of the same.

This motion was discussed *pro* and *con*. Several members were of the opinion that this was a matter that could be best arranged by members themselves as occasion required. Motion was finally carried. Dr. Cooper then moved that the Chair appoint a committee of three on the substitute proposition, to report at the next meeting. The Chair appointed, as such committee, Drs. Cooper, Reagan, and Doty.

Dr. J. Payne Lowe spoke in favor of the Association paying the expense of sending specimens to the State Laboratory for bacteriological or other examination when the Association received the benefit of such examination. Upon motion of Dr. Reagan the Association authorized such expense to be paid out of the treasury when, in the judgment of the President, the case was of such a nature or character as to warrant the same, but such expense was only to be allowed upon the written order of the President.

The Treasurer, Dr. M. A. Pierce, reported all bills paid and $13 balance in the treasury.

The delegates to the annual meeting of the Veterinary Medical Association of New Jersey, held at Trenton, January 8, 1903, made the following report:

" Your delegates would report that the Passaic County Veterinary Medical Association was represented at Trenton by Drs. Pope, J. Payne Lowe, Fredericks, and President William Herbert Lowe.

"The sessions were well attended by leading members of the profession from all parts of the State, by several State officials, as well as by a number of prominent veterinarians from neighboring States. Among the visitors from Pennsylvania were Drs. Leonard Pearson, Dean of the Veterinary Department of the University of Pennsylvania, and W. Horace Hoskins, editor of the JOURNAL OF COMPARATIVE MEDICINE AND VETERINARY ARCHIVES; from New York, Dr. Veranus A. Moore, Professor of Comparative Pathology and Bacteriology at the New York State Veterinary College, Cornell University, Ithaca; and from New York City, Drs. George H. Berns, E. B. Ackerman, Robert Dickson, Robert W. Ellis, James L. Robertson, and Roscoe R. Bell, editor of the *American Veterinary Review*, the three latter being professors in the New York American Veterinary College, New York University.

"The morning session was given over to the transaction of routine business, the election of officers, and the delivery of the President's address. There were many salient points in the address. Dr. Lowe recommended the establishment at Trenton of a State Bureau of Commission of Animal Industry, with a Chief Veterinarian as State Veterinarian. With such an organization the veterinary sanitary work of the State could be more efficiently and economically administered than by a number of separate bureaus working independently without professional directorship, as at present. Dr. Lowe said in his address that a layman or veterinarian could with just as much propriety attempt to direct the official work of the medical profession in the State as a layman or an M. D. has to undertake to direct the veterinary work of the State.

" A Conference Committee was appointed to confer with the officers of the State Board of Health, the State Board of Agriculture, the State Tuberculosis Commission, and others concerned, with a view of adopting a plan satisfactory to all concerned, as recommended by the President. As soon as preliminaries are arranged it is proposed to take steps to secure the necessary legislation.

"The afternoon session was devoted to the reading of papers and discussions. Professor Veranus A. Moore read an excellent paper on 'Etiology and Prevention of Infectious Diseases of Animals,' illustrated by the stereopticon.

" A paper on the 'Relation of State Boards of Veterinary Examiners to the Teaching Schools, the Profession, and the State,' by Drs. T. B. Rogers and William Herbert Lowe, was read and discussed.

" Dr. Leonard Pearson gave some valuable data based on experiments made by him on the immunization of cattle against tuberculosis.

"The Veterinary Medical Association of New Jersey will hold its semi-annual meeting at the United States Animal Quarantine Station at Athenia, on the Newark and Paterson branch of the Erie Railroad, and on the Boonton branch of the Delaware, Lackawana and Western Railroad, on Thursday, July 9, 1903, which will give members of the profession an excellent opportunity not only of seeing fine specimens of imported stock, but of learning something of the method and system of inspection and quarantine as conducted by the Federal Government."

The report of the delegates was received and it was moved and carried by unanimous vote that the Passaic County Veterinary Medical Association approve and endorse the recommendations of President William Herbert Lowe made in his recent address before the Veterinary Medical Association of New Jersey, at Trenton, for the establishment of a State Bureau or Commission of Animal Industry with a Chief Veterinarian as the State Veterinarian of the State of New Jersey, and that a committee be appointed by this Association to aid the committee of the State Association in this important undertaking. The Chair appointed, as such committee, Drs. Pope, Fredericks and J. Payne Lowe.

The Association learned with regret of the illness of Dr. John Kehoe, and, upon motion of Dr. Machan, Dr. J. Payne Lowe was appointed a Committee of one to call upon Dr. Kehoe at his home.

The President appointed Dr. Reagan Essayist for the March meeting. The subject of his paper is "Poisoning in the Dog." On motion meeting adjourned at 10.30 P. M.

D. MACHAN.
Secretary *pro tem.*

The regular monthly meeting of the Passaic County Veterinary Medical Association was held at 169 Paterson Street, Paterson, N. J., on Tuesday evening, March 3, 1903.

The President being absent, his brother, Dr. J. Payne Lowe, was requested to take the chair. He called the meeting to order at the usual hour and upon roll-call a quorum was declared present.

The minutes of the last regular meeting were read and approved.

Dr. Cooper reported progress for the Committee on Substitute.

Dr. J. Payne Lowe reported that Dr. Kehoe was much better and out again.

Dr. Cooper raised the question as to whether glanders was on the increase or decrease in this county: which was discussed by the members present, each giving his individual experience.

The members then listened to a most creditable paper on "Poisoning in the Dog," by Dr. Reagan. Dr. Reagan was given a vote of thanks, and it was ordered that a type-written copy of his paper be sent to both veterinary periodicals for publication. Dr. H. K. Berry was appointed Essayist for the April meeting. On motion meeting adjourned at 10.30 P. M.

WM. J. FREDERICKS,
Secretary *pro tem.*

The regular monthly meeting of the Passaic County Veterinary Medical Association was held at the office of Dr. Wm. Herbert Lowe, corner of Paterson and Van Houten Streets, Paterson, N. J., on Tuesday evening,

April 7, 1903. The meeting was called to order at the usual hour by First Vice-President, Dr. David Machan.

The minutes of the March meeting were read and approved.

*Unfinished business.* Committee on Substitute reported progress.

*New Business.* Dr. Cooper reported that a certain practitioner was charging less than the schedule rate. On motion this matter was laid over until the next meeting.

Dr. H. K. Berry, the Essayist, being absent, several interesting cases were reported and discussed and the meeting turned out to be an interesting and profitable one. On motion meeting adjourned at 10.30 P. M.

J. PAYNE LOWE,
Secretary *pro tem.*

---

### MEETING OF THE VETERINARY MEDICAL ASSOCIATION OF NEW JERSEY.

The semi-annual meeting of the Veterinary Medical Association of New Jersey will be held at the United States Animal Quarantine Station for the port of New York, located at Athenia, N. J., on Thursday, July 9th.

Veterinarians of neighboring States are cordially invited to attend. Athenia is on the Newark and Paterson branch of the Erie Railroad, and also can be reached via the Boonton branch of the D., L. & W. R. R.

---

### THE ILLINOIS STATE VETERINARY MEDICAL ASSOCIATION.

The twenty-first semi-annual meeting of this Association was held in Urbana, February 17th, at Morrow Hall, State Agricultural Building.

Meeting called to order at 10 A. M.

Members present:

Drs. W. W. Giles, of Gilman; E. J. Firt, of Havana; F. H. Ames, of Canton; D. McIntosh, of Champaign; T. W. Corkery, of Urbana; H. I. Stringer, of Watseka; L. C. Tiffany, of Springfield; F. C. Grayson, of Paxton; C. S. Hayward, of Mattoon; E. T. Quitman, of Chicago; G. G. Ratz, of Red Bud; F. H. Barr, of Pana; W. J. Martin, of Kankakee; H. A. Pressler, of Fairbury; D. E. Kinsella, of Chillicothe; Geo. B. Jones, of Sidell; C. C. Mills, of Decatur; A. H. Baker, of Chicago; Geo. S. Frye, of Naperville.

New member elected:

Dr. Andrew Robertson, of Mt. Carmel.

Minutes of last meeting were read and approved.

Report of Treasurer showed balance on hand of $88.69.

Bills allowed:

| | |
|---|---|
| For printing 2000 envelopes | $6.00 |
| For printing 1000 letter-heads | 4.50 |
| For printing 500 Constitutions and By-laws | 20.00 |
| For printing 40 programmes | 4.00 |
| For stamps | 4.00 |
| For express | 60 |
| For Secretary's fees | 10.00 |
| Total | $49.10 |

The following papers were read and discussed:

"Gastrointestinal Catarrh." By Dr. F. H. Barr, of Pana.

"Veterinary Obstetrics." By Dr. W. J. Martin, of Kankakee.

"Fraternalism in Veterinary Science." By Dr. T. W. Corkery, of Urbana.

"Analogous Foot-and-Mouth Disease." By Dr. C. C. Mills, of Decatur.

"Azoturia and its Treatment." By Dr. F. H. Ames, of Canton.

"The Treatment of Tetanus by Excessive Doses of Potassium Bromide." By Dr. D. McIntosh, of Champaign.

Under this last topic Dr. McIntosh reported the recovery of 23 cases of tetanus, each of which received from 3½ to 5 pounds of potassium bromide in their drinking-water, covering a period of three to five days. He stated that he encouraged the animal to drink excessive amounts of this drug, and invariably when he had received from 3½ to 5 pounds perfect relaxation of the muscles occurred, and recovery took place. There seems no danger in pushing the drug, and he aims to get this amount into the animal in as short a time as possible, usually from two to five days.

It being understood that a bill had been presented to the Legislature amending our present bill so that registration might again be opened for a period of six months to those quacks who had not availed themselves of securing a license to practise, the following resolution was presented by the Committee on Legislation, and passed.

*Resolved*, That it be the sense of this Association that we are strongly opposed to the passage of any amendment to the present Veterinary Bill.

Moved and seconded that the Secretary notify all members and secure their co-operation to defeat the passage of the amendment.

A vote of thanks was given the Trustees of the Illinois College; also to Dr. McIntosh for the use of the hall and other courtesies.

Adjourned to meet in Chicago at the call of the President.

W. H. Welch,
Secretary.

---

### CENTRAL CANADA VETERINARY ASSOCIATION.

The second meeting of the Central Canada Veterinary Association was held in the Committee Room of the City Hall, Ottawa, on Monday evening, April 13th, President Harris in the chair.

The meeting was well attended, those present being Drs. Fisher and McGregor, Carleton Place; Higginson, Rockland; McGuire, Cornwall; Thacker, Renfrew; Young, Almonte; Young, Cobden; Young, Merrickville; Lynchke, Carp; Allen, Brockville; Haworth, Eganville; Rutherford, Harris, Higgins, Hollingsworth, White, and Boucher, of Ottawa.

The following new members were elected: J. J. McGregor, Carlton Place; C. W. J. Haworth, Eganville; W. G. Gilpin, Ottawa; Charles Thacker, Renfrew, and J. Massie, of Kingston.

The Constitution and By-laws as prepared by the committee appointed at the previous meeting were adopted, with few changes. The Constitution and By-laws provide rules for the conduct of business, a Code of Ethics regulating professional conduct, qualifications for membership, and a Council to whom matters pertaining to the welfare of the Association are referred.

A resolution expressing sympathy with Dr. A. E. James, a member of the Council, in his recent illness, was passed.

The coming meeting of the A. V. M. A. was referred to by Dr. J. G. Rutherford, who stated that while it was desired that as many as possible should avail themselves of the privilege to become members, this was not necessary, and they would be heartily welcomed as guests at the meeting in September, when the veterinary profession would practically own the capital city of Canada.

On motion of Dr. Hollingsworth, of Ottawa, seconded by Dr. McGuire, of Cornwall, it was voted that the Central Canada Veterinary Association give the sum of one hundred dollars toward the entertainment of the A. V. M. A. at its coming meeting.

Dr. J. G. Rutherford, Chief Veterinary Inspector to the Dominion, gave a very interesting and instructive talk upon the present method of dealing with glanders in this country. He stated that his idea in presenting in detail the present method of dealing with this disease was to acquaint the members of the profession (who in the past had attempted to manage outbreaks under the Provincial laws) with a quicker and better solution of the problem, namely, that of reporting them to the department which had the authority to take the matter in charge without the delay necessitated by summoning witnesses and bringing delinquents before magistrates, which course has up to the present been necessary. He also stated that the practical results of the present methods of dealing with outbreaks of glanders would, in all probability, be presented to the meeting in September.

The report of a case of phimosis was given by Dr. Lynchke, of Carp, which evolved numerous comments and considerable discussion.

On motion of Dr. McGuire, the meeting adjourned.

CHAS. H. HIGGINS,
Secretary.

---

### PENNSYLVANIA STATE VETERINARY MEDICAL ASSOCIATION.

(Concluded from page 267.)

Dr. S. J. J. Harger, as Chairman of the Committee on Animal Husbandry, presented the following report:

*Report of Committee on Animal Husbandry.*

The Committee on Animal Husbandry was created two years ago, and only a few reports have been submitted to you. We therefore have no well-trodden paths pointed out by our predecessors to follow; besides, it is only recently that special interest has been awakened upon the relation between animal husbandry and the veterinary educator and practitioner. The specialization of this work from such a standpoint being still immature, we will discuss some of its generalities and perhaps some details.

What we understand by animal husbandry is not a commonplace undertaking of a nature that any one who has a fondness for animals can follow, but one that requires certain qualifications. One needs some training in comparative anatomy, zoology, physiology, biology, chemistry, and botany, a superstructure such as is given in a good agricultural school.

In the language of the schools, animal husbandry is called *zootechnics*—an industrial exploitation of the domestic species. It studies the living animal, not in disease but in health, in a physiological sense. We operate upon the animal to ameliorate his condition, increase his products, intensify the economic functions, such as milk, beef, wool, work, speed, improve the type, and create new breeds. We make use of any operation that will enhance his value.

This requires a knowledge of the principles of heredity, sexual selection for different purposes as modified by the nature of the stock at our command, rearing of the young, feeding for special purposes, the exterior or conformation, animal dynamics, fitting of the harness, shoeing, influence of soil and climate.

In all this work the veterinarian may play an important rôle. In these days of the survival of the fittest he must be able to do more than diagnose influenza or resect the cunean tendon. The practice of his profession is not incompatible with the legitimate exploitation of the domestic species in the breeding or the dairy farm. He may not be a practical breeder or an animal economist in its fullest sense, but from his training and knowledge of soundness and heredity he becomes upon some points the natural expert. He is acquainted with the conditions preserving health, the physiological functions, and how to stimulate these to perform their maximum work.

Unfortunately very few of the veterinary schools give the necessary instruction upon this subject, and do not have the facilities for practical teaching. It would be fortunate if schools could add to their clinic room a farm on which animals could be exploited. Information can be gained from text-books, bulletins issued by the experimental stations, visiting, breeding, and dairy farms, and horse shows to study the characters of the breeds and their management. The veterinarian should be a better judge of breeds than horsemen, who are experts by virtue of their millions.

The necessity for the amelioration of our domestic species is very evident. Live-stock constitutes one of our largest industries, representing several billions of dollars. The yearly export of farm animals amounts to $50,000,000 and that of animal products $180,000,000. One-fifth of our total annual exports consists of animals and all products and articles manufactured therefrom.

The national prosperity of a country is closely related to the state of its agricultural pursuits and its live-stock. Mohammed (632-57 B.C.), who established the first Arab stud, recognized the need of improved horses. What has been accomplished since can be seen by comparing the Asiatic and African cows, which barely give enough milk to suckle the calf, or the native ox of a century ago, with a Jersey or a Hereford.

Modern animal husbandry tends to specialization of aptitude. Economic conditions point to specialization for certain purposes to the exclusion of all others. The dual-purpose cow, excepting the milking families of the Shorthorn, is fast disappearing. The law of organic balance antagonizes the intense development of more than one economic function in the same individual. The beef, milch, draft, speed, mutton, and wool types have their special characteristics, and thus the creation of many breeds. The animal body is *pliable.* It responds to its environments, feeding, and methodical exercise, which, if the proper conditions be supplied, develop peculiarities and variations that can be still further intensified by judicious breeding.

The Jersey has been especially pampered and fed to produce butter for the London market and the Ayrshire to supply milk for the cheese factories of the surrounding counties. The Dutch (so-called Holstein-Friesian) cow, the most copious milker of all breeds, has been exploited in the same sense for two thousand years. Collings saw in one of his half-bred Dutch calves unusual form and precocious development, and thus laid the foundation of the Shorthorn. Anomalous variations, called "Sports," which are hereditary, sometimes appear and develop into breeds characteristic of distinct economic value. Among such we have the niata, or bulldog-nosed cattle of South America, the polled Durham, polled Angus, corkscrew-tailed French bulldog, and the silken-wooled Mauchamp merino sheep. Fossil deposits of hornless cattle have never been discovered.

One of the greatest problems is the breeding question. Upon it depends the creation of superior families and breeds. Too much emphasis cannot be placed upon the idea of breeding for a purpose, having in mind a definite economic function, type, or breed to be produced, and then selecting the producer accordingly, keeping in view the adaptability of the animal to the location and the market demands. Every animal should be raised with the thought of being well adapted for a certain purpose. This gives him real value. Promiscuous breeding with the "travelling stallion," selected because of its small stud fee irrespective of type or soundness, has destroyed types and brought our live-stock into a state of disordered variation. Vicious crosses should be avoided. Sire and dam should be as much as possible of the same type. If both parents are of sound constitution this may be supplemented by consanguinity or in-breeding. Two unlike types may become harmoniously blended, but this requires judgment and time. The farmer's son deceives himself in expecting to raise a trotter from the common farm mare and a standard-bred stallion standing in the community. This stallion has in this way been harmful to the horse industry in many localities. Such methods have placed upon the market a surplus of unsalable, no-purpose horses. Animals adapted for a special purpose are always in demand. American horses in Europe can be recognized, it is claimed, by a want of uniformity in type.

The various countries of the world have variously contributed to the specialization of the domestic species. Scotland has given us the Clydesdale horse under the patronage of the Highland Agricultural Society, which placed at the disposal of farmers Clydesdale stallions. England, the greatest breeding country in the world, created the Shire horse, and winners of the prizes offered by the Royal Agricultural Society are much sought after as breeders; also, the thoroughbred and hackney. Among cattle the Jersey, Guernsey, polled Durham and Angus, and the latter of the Dutch ancestry from across the English Channel, combined with the native cows of the Teeswater. The Shorthorn, more than any other, has been the potent factor in ameliorating American cattle. France, with its government assistance, developed the well-known Percheron and the Anglo-Norman or French coacher, the latter the product of the old Norman mares and the thoroughbred stallions; also, two other breeds, the Brittany and the Boulonnaire. Some of the latter breed are sent into the valley of the Perche, there "percheronized" and sold to unsuspecting foreigners as Percherons. Germany produces the Frakener and the Mecklenberg, Oldenberg, and Hanoverian coachers; Russia, the Orloff trotter, and Holland and Belgium and Dutch and Belgium draft horses› and the Dutch or Holstein cow; in fact, Holland, Belgium, and adjoining France have from time immemorial been the home of the draft horse in his wild state, which here has been taken into other countries and transformed into the different draft breeds. The only distinctly American breeds are the polled Durham and the trotting horse.

In the United States we have a great expanse of country, fertile soil, and other natural resources favorable to stock-raising. Our native stock had to be improved by importations of pure breeds from Europe, whose prepotency soon became manifest. The Texas steer is much superior to that of a few decades ago from the introduction of Shorthorn and Hereford bulls. We are now competing with England to create a South American market for our pure-bred cattle. Our Jerseys and Guernseys are larger and more copious milkers than those of their native islands. The mustang has passed away. The range-bred horse is of higher class and breeders are using care in selection.

Nearly all European nations have governmental studs and officially give encouragement to the breeding of live-stock, especially horses. France, perhaps, leads them all upon this point, and it is to this, very largely, that she owes the production of her renowned breeds. This government maintains studs under the surpervision of an Inspector-General, where stallions are raised. Stallions are also kept at the stud remounts in each department, and placed at the disposal of farmers and breeders for a nominal fee. Stallions owned by citizens are licensed, and are divided into three classes—authorized, approved, and certified. The first is the highest and the last the lowest recommendation. These stallions are examined by a commission composed of the Inspector-General or his deputy and a breeder and a veterinarian appointed by the prefect of that department.

In this country national regulations cannot at this time be expected. Our country is so vast and the breeding interests so large that it would require a well-organized bureau to assume control, and our government is averse to multiplying its bureaus. The most that we can expect might be State control under the Department of Agriculture of the State. I believe that such a step would do much to enhance the breeding interests, and that this idea should be encouraged until its adversaries are reconciled. A registration law pertaining to breeding animals is, I believe, in existence; but it is evidently not what is desired and has never been enforced.

An industry of recent origin in some of the central western States—Illinois, California, Oregon, and Montana—is the breeding of Angora goats imported from Persia. A complete description is found in the Annual Report of the Bureau of Animal Industry, 1901. This animal subserves a threefold purpose: It furnishes good mutton, which can take the place of sheep mutton; it produces wool, or mohair, manufactured into expensive fabrics, and it clears the unoccupied land of underbrush, in which it seems to thrive, and prepares the land for grazing and cultivation. To one who understands the industry and has a little capital this business offers inducements.

Brazil, a few years ago. commenced to breed zebroids. These are hybrids between the mare and the male zebra, and in their general form resemble the zebra, but are much less striped.

Feeding constitutes an important division of animal husbandry. Feeding, or functional gymnastics of the digestion tract, as it is called, is one of the most potent factors in producing useful variations in any part of the body. Intensive alimentation stimulates all the nutritive functions. It produces early maturity, or precocity. The body becomes more compact and short-legged from the early coalescence of the ossific centres of the long bones. In the cow it intensifies the milk secretion. Young animals, especially those intended for breeding purposes, should be well fed; when poorly fed they mature later, become more leggy and less symmetrical. The stock of a locality should be adapted to the feeding conditions. The larger and more precocious breeds require better management and feeding; the smaller, such as the Jersey, Guernsey, Ayrshire, and the little Brittany, which are the poor man's cows, are better foragers, and better adapted to the poorer pastures and severe winters of Canada and New England.

When the production of a quart of milk under good sanitary conditions costs a fraction more than two cents, economic feeding is an important item to the dairyman who sells his milk wholesale, and the loss incurred by injudicious feeding will reduce his profits. The discussion of the normal or balanced and the unbalanced rations is not idle talk. The preservation of the nutrition ratio, by which we mean the proportion between the digestible proteids on the one part and the combined digestible hydrocarbons (fats) and carbohydrates (starch and sugar) on the other is the basal factor in rational feeding. Too narrow or too wide a ration means a waste of feed. The composition of the ration, besides, should vary according to age and purposes of feeding. The heavy milker requires a more narrow ration—that is, more albumin than the poor milker. The race-horse requires a more narrow ration than the draft-horse. The young, growing animal, which builds up the albuminous tissues of the body, needs a more narrow ration than the adult. Any number of ration combinations can be made in these proportions, according to the food-stuffs at hand and their cost. The food standards cannot always be accepted verbatim, but their underlying principle is a good guide. One must determine what food-stuffs to grow in order to maintain the largest number of animals on the smallest acreage. Soiling, ensilage, and alfalfa recommend themselves because of their heavy yield of forage.

Pennsylvania is a remarkable State for two reasons: its extensive agricultural pursuits and its unequalled mineral products—coal, iron, and oil. The richest agricultural counties are found in the Cumberland Valley, along the south and the southeast; the west, along the Delaware River; the northeast, the northwest, and the southwest. The central counties, extending to the north and northwest, are mountainous and less productive.

The sheep and wool industry has deteriorated. In 1890, 1,612,107 pounds of wool were produced; in 1900, 959,483 pounds, a reduction of 40.5 per cent. Breeders have abandoned sheep-raising, principally on account of the ravages of dogs. Sheep, after being chased by dogs, are prone to abortion, and fatten with difficulty. There is a large area of unproductive land in the central part of the State well adapted for this purpose. A bill is at present before the Legislature requiring all dogs running at large to wear a collar bearing the name and address of the owner. All dogs not so identified can be shot with impunity.

The swine industry is but little developed.

In view, therefore, of many undeveloped resources and the consumption of animal products in excess of production, we see the necessity of introducing modern methods of agriculture and stock-feeding and of improving our grade breeds, in order to equalize the supply and demand and increase the revenue from the land.

S. J. J. HARGER, V S.,
Chairman.

The Committee on Meat Inspection made no report.

The reports of committees having been finished, the subject was then opened for discussion.

The discussion was opened by Dr. Hoskins, who was especially well pleased with the report of the Committee on Intelligence and Education. He wished to add, in addition to the report made by this committee, our recognition of the excellent work done by the Bureau of Animal Industry at Washington under the direction of Dr. Salmon; and also the unequalled work done by our State Live-stock Sanitary Board through the efforts of Dr. Pearson.

Dr. John L. Bradley commended the report of Dr. Harger. He differed with the chairman in reference to crossing pure-bred stallions on native mares. This may not give as good results as where pure-bred mares are used, but he feels sure that the type of horses in the community would be improved by using pure-bred stallions with the native mares.

Dr. Kooker emphasized the importance of selecting pure-bred males with good individual qualities, and as free as possible from defects. By judicially crossing these with females possessing the same qualities good results can be expected. He is a strong believer in heredity and cited several illustrations. One case in particular was a mare stinted to the "Duke of Wellington." The result was a filly that developed three ringbones at the age of three months. Her next foal by the same sire developed four ringbones at about the same age, and was destroyed for this reason. The sire was blamed for these faulty foals, yet no other colts from him showed this defect. This same mare was then stinted to the gray stallion "Pilot," owned at Newton, N. J., and the produce developed three ringbones inside of three months. It was found on investigation that this mare's dam had three ringbones.

Dr. Harger said he believed in hereditary defects to a certain extent. According to observation, spavin is more often transmitted than ringbone. He thinks that not only hocks, or fetlocks, of bad conformation may be transmitted, but that the substance of the bone, if of a poor quality, may be transmitted also.

Dr. J. C. McNeil believes that faulty conformations are transmitted, while accidental defects, as blindness, ringbones, spavin, etc., when they are known to be due to accidental causes, should be no bar to breeding.

It is the opinion of Dr. A. F. Schreiber that the care given to animals has as much or more to do with the offspring as hereditary tendencies do; yet, other things being equal, animals with a pedigree should be chosen, for, as a rule, like produces like.

Dr. H. B. Felton cited a herd of cows that had been under his observation for several years. These were ordinary native cows to start with, but care has been exercised for a number of generations in selecting pure-bred Guernsey bulls of good milking strains to cross with these cows. At the present time this herd has the appearance of thoroughbred Guernseys.

Dr. John L. Bradley again took the floor and concurred with the views of Dr. Felton. He believes that where people cannot afford to have pedigreed stock all through, they should at least choose males of the best possible breeding. People should first decide for what purpose animals are to be used. If it be cows for dairy purposes, use the cows already on hand and get thoroughbred Guernsey or Jersey bulls, and in time good milkers will be produced.

Dr. Hoskins spoke of the constant effort necessary in order to keep any breed or characteristic from losing its identity. He commended highly the report made by Dr. Harger for the Committee of Animal Industry, and urged on the members the necessity of studying the subject of breeding more carefully; that we should take more interest in the work done by the National Breeders' Association and live-stock exhibitions.

Dr. J. F. Butterfield has had the advantage of being a veterinarian as well as a breeder of pure-bred animals. He is a firm believer in the subject of heredity, and believes that peculiar traits may be transmitted for hundreds of generations. He cited as an illustration of this fact shying in horses, which he says may be due to the fear that horses had of wild beasts in the remote past.

The subject of discussions on the reports of committees being closed, the meeting adjourned until Wednesday morning at 10 o'clock.

THURSDAY, MARCH 4, 1903.

The first order of business was the report of the Board of Trustees. They reported favorably on the resignation of Dr. J. C. Kingsland, of Canton, Bradford County. This was regularly adopted by the Association.

The application of Dr. S. C. Weamer lacked the necessary vouchers. The secretary was instructed to return his application and request Dr. Weamer to be present at the semi-annual meeting, when his application would again be considered.

The Corresponding Secretary read a letter from the widow of the late Dr. Reinhart, requesting the Association to dispose of a pair of obstetric forceps for her.

Correspondence was also read from E. W. Powell, Bryn Mawr; J. W. Moore, Myerstown; C. R. Good, Lockhaven; W. A. Meredith, and A. J. Tupler.

*Corresponding Secretary's Report.*

Dr. M. Moriarity, of Gettysburg, was present and made a verbal report for Adams County.

The following written reports were read and filed: Dr. J. C. Kingsland, Bradford County; Dr. H. W. Turner, of Bucks County; Dr. W. B. Prothero, Cambria County; Dr. W. H. Fry, Centre County; Dr. James B. Rayner, Chester County; Dr. George Magee, Fayette County; Dr. M. J. Collins, Lebanon County; Dr. A. W. Weir, Mercer County; Dr. J. F. Butterfield, Susquehanna County; Dr. G. B. Jobson, Venango County; Dr. W. P. Bagnall, Warren County; Dr. W. J. Waugh, Washington County. Dr. L. E. Meade made a verbal report for Wyoming County. He has the distinction of being the only member of the Association in the County.

Dr. H. P. Eves, of Wilmington, Del., was called to report on veterinary matters in his State. He responded by mentioning the following diseases as prevalent in Delaware: Rabies, hog cholera, anthrax, tuberculosis, forage poisoning, and straw poisoning.

There is a movement at present in his State to secure the necessary laws in reference to regulating the practice of veterinary medicine. At the present time there are practically no laws on this subject.

Dr. Leonard Pearson was called to explain what was being done at Harrisburg in reference to legislation that is of interest to veterinarians especially. He mentioned a number of measures that had been recently agitated or introduced. A bill was recently introduced to allow castrators to register as second-class veterinarians. It is not likely to pass.

A bill has been introduced which aims to prevent the spread of rabies and to suppress the damage done to sheep by dogs. This requires that all stray dogs can be destroyed or quarantined. He thinks that this bill will become a law.

The decrease in the sheep industry in this State is supposed to be due in a large measure to the ravages of dogs.

A bill has also been introduced to prevent the sale of veal calves before they are four weeks old. Such calves should be tagged and put under the control of the State Live-stock Sanitary Board.

A bill to make it possible for butchers to receive pay for tubercular cattle at five cents per pound and not over twenty-five dollars per head. Such meat is to be inspected and the inspector is to be governed by the rules of the Bureau of Animal Industry, or by rules satisfactory to the State Live-stock Sanitary Board.

A bill requesting the appropriation of $3000 to make the necessary investigations in reference to immunizing cattle against tuberculosis has been introduced.

An effort is also being made to obtain an appropriation of $100,000 to assist the University of Pennsylvania in building a new veterinary department.

A photograph of the Association was taken by a representative of the Philadelphia *Press*, after which the Association adjourned for lunch.

The following papers were read and filed: Dr. J. B. Rayner presented two papers, one "Paralysis of the Bowel in a Horse," the other a "Peculiar Case of Tetanus." "The Origin of the 2.10 Trotter," by Dr. Charles Lintz. "Snags that a Veterinarian May Run Against," by Dr. W. S. Kooker. "Requirements for a First-class Dairy," Dr. J. F. Butterfield. "Horse-show Responsibilities," Dr. C. J. Marshall. "A Rare Case," Dr. M. J. Collins. Owing to the lack of time these papers were not discussed.

The embryotomy shears belonging to the late Dr. N. E. Reinhart were auctioned off by Dr. Pearson to the highest bidder. They were purchased by President Rhoads for the sum of four dollars.

The following resolutions were unanimously adopted and ordered spread on the minutes:

*Resolved,* That inasmuch as the veterinary profession of our State was well supported during the past four years under the administration of the ex-Governor, Hon. William A. Stone; therefore, be it

*Resolved,* That we, the PENNSYLVANIA STATE VETERINARY MEDICAL ASSOCIATION, in convention assembled, do most cordially express our sincere appreciation of the kindly interest and assistance he always afforded us.

*Resolved*, That we heartily endorse the Sproul Good Roads bill with the Roberts amendment now pending in the Legislature, and urge the active support of every member of the profession to this proposed legislation.

*Resolved*, That we commend most heartily the well-directed efforts of our President, Rev. Dr. W. L. Rhoads, in the interest of good roads throughout the State, and acknowledge our indebtedness to him for his earnest and valuable work.

*Resolved*, That we heartily approve and deeply appreciate the retention by the Governor, Hon. Samuel W. Pennypacker, of our present State Veterinarian, Dr. Leonard Pearson.

*Resolved*, That we cordially recommend to the Governor the name of Dr. M. E. Conard for the position of Dairy and Food Commissioner.

WHEREAS, The proper education of those who enter the veterinary profession is of the utmost importance to the people of the State; and

WHEREAS, The work of the Veterinary Department of the University of Pennsylvania has been of such a high character as to meet the approbation and commendation of the veterinary profession and all who are interested in higher education; therefore, be it

*Resolved*, That we urge the members of the State Legislature to support the bill appropriating one hundred thousand dollars ($100,000) toward the erection of a suitable building for the veterinary department of the State University.

WHEREAS, Clean and honest trials of speed are of great value as a means of developing the quality of horses, and are most interesting and instructive as exhibits; and

WHEREAS, Horse-racing receives its best development in the direction of wholesome sport, and is most advantageous to breeders in countries and States where it is under proper governmental control and supervision, while, on the other hand, it is not conducted in the interest of pure sport nor in the interest of horse-breeders in States where there is not some oversight from a high and impartial tribunal; be it

*Resolved*, That we, the PENNSYLVANIA STATE VETERINARY MEDICAL ASSOCIATION, recommend the establishment of a State Racing Commission, to be composed of men interested in the improvement of horses, to be selected by the Governor, and to be empowered to license and supervise race courses and race meetings, to the end that horse-racing may not be merely tolerated without legal status, but shall be placed on a fixed foundation and shall be honestly conducted, free from gambling. In this way horse-breeding and the improvement of horses may have fixed standing and definite support.

*Resolved*, That we most earnestly recommend that the incoming officers will insist upon the chairmen and members presenting their reports in writing, or that the Association shall employ a stenographer, that these valuable reports may be properly filed and greater publicity given to the same.

WHEREAS, The great benefits enjoyed by the entire people of our State through the valuable investigations, the great protective measures afforded through the work of the laboratory of the State Live-stock Sanitary Board; and

WHEREAS, The scope of this work has been very much circumscribed by the limited means at their command, and the greater need at this time of more extended investigations of other diseases whose origin is still undetermined, and for which we are without adequate means of defence or protection; therefore, be it

*Resolved*, That we most urgently recommend a favorable consideration by our State legislative bodies of the asked-for appropriation of $30,000 for the extension of this work.

WHEREAS, We believe that the best interests of our Association will be conserved by a change in our by-laws relating to the election of officers, and we recommend that the Board of Trustees will prepare and submit for the consideration of the Association, at the semi-annual meeting, such an amendment as will provide for the nominations for all elective offices to be made on the first day and the election on the succeeding day thereafter.

The officers for the ensuing year were introduced and installed in their new positions.

Dr. W. L. Rhoads made a motion, which was adopted, that the selection of a place for our semi-annual meeting be left to the Board of Trustees.

Dr. J. C. McNeil made a motion, which was regularly adopted, that a vote of thanks be extended to our local Committee of Arrangements, composed of Dr. W. S. Kooker, S. J. J. Harger, and H. B. Cox; also, to the Reception Committee, composed of C. J. Marshall, B. M. Underhill, and E. M. Ranck.

Meeting adjourned at 5.40 P.M.

C. J. MARSHALL,
Secretary.

---

### PENNSYLVANIA STATE BOARD OF VETERINARY MEDICAL EXAMINERS.

The semi-annual meeting of the Board convened at Philadelphia, Pa., December 15 and 16, 1902. There were two applicants before the Board: Dr. B. E. Nevel, Dover, N. J., and Dr. P. J. Purcell, Bradford, Pa., both having been before the Board at previous examinations. Drs. Nevel and Purcell were granted the license of the Board.

At the meeting of the Board on December 16th Dr. Hoskins reported on the successful prosecution of P. G. White, of Scranton, Pa.; also, on the prosecution of C. E. Hyle, of York, Pa., which was unsuccessful, owing to the decision of the Judge, that as C. E. Hyle had practised for twenty-one years, and for a period of more than twelve years in this Commonwealth, he stated in his charge to the jury the law of 1895 abrogated those of 1889 and 1891, and directed the jury to acquit the defendant.

Dr. Hoskins reported a contribution of two hundred dollars ($200) as having been received from the Pennsylvania State Veterinary Medical Association in August.

Bills were reported for the prosecution of Messrs. White and Hyle, and bills for expenses in attendance at the meeting, all of which were ordered paid.

Approval of the State Association recommending reregistration over the State of all veterinarians, and that the Board co-operate with the Association and profession for the accomplishment of this purpose; that the Secretary write Dr. Jobson relative to the proposed legislation and confer with the Board.

On motion, the meeting adjourned.

W. HORACE HOSKINS,
Secretary.

